# Validation of mixed reality in planning orbital reconstruction with patient-specific implants

**DOI:** 10.1038/s41598-025-85154-4

**Published:** 2025-01-15

**Authors:** K. Dubron, E. Shaheen, R. Jacobs, C. Politis, R. Willaert

**Affiliations:** 1 Department of Oral and Maxillofacial Surgery, University Hospitals Leuven, Leuven, Belgium; 2 OMFS IMPATH Research Group, Department of Imaging & Pathology, University Hospitals Leuven, Leuven, Belgium; 3 Department of Dental Medicine, Karolinska Institutet, Stockholm, Sweden

**Keywords:** 3D virtual planning, Mixed reality, Virtual reality, Augmented reality, Preoperative planning, Traumatology, Fracture repair, Three-dimensional imaging, 3-D reconstruction

## Abstract

This study aims to evaluate and compare the usability and performance of mixed reality (MR) technology versus conventional methods for preoperative planning of patient-specific reconstruction plates for orbital fractures. A crossover study design was used to compare MR technology with conventional three-dimensional (3D) printing approaches in the planning of maxillofacial traumatology treatments. The primary focus was on user-friendliness and the accuracy of patient-specific reconstruction planning. Secondary outcomes included investigating time differences between the two approaches and evaluating the potential effects on the learning curve. Participants were asked to complete questionnaires assessing various aspects, such as visualization, interaction, segmentation, treatment planning, and evaluation. Objective endpoints were evaluated blindly, while subjective endpoints were analyzed through a double-blind process. The total workflow time for MR technology was significantly shorter compared to the conventional method. Moreover, treatment planning using MR was significantly more accurate (*p* = .028), with participants reporting a higher mean global satisfaction score compared to the conventional group (80.6% vs. 72.5%). This study sheds light on the potential benefits of employing MR technology in maxillofacial orbital reconstruction. This preoperative method allows for faster and more precise design of patient-specific implants for orbital reconstruction, potentially leading to improved long-term cost-effectiveness.

## Introduction

Traumatic injuries to the orbit are a common occurrence, presenting significant challenges due to the intricate three-dimensional (3D) structure of the orbit during preoperative reconstruction^1^. The limited orbital space adds further complexity during intraoperative reconstruction. The current focus of surgical management is on reconstructing the bony anatomy and supporting soft tissue structures by restoring the original orbital volume^2–4^. In order to highlight the significance of preoperative planning, a shift in focus is necessary. This calls for the use of additional methods or advanced technology to aid surgeons in their preoperative planning^5–8^.

Mixed reality (MR) is an innovative dimension that combines virtual reality (VR) and augmented reality (AR) to create a unique blend of the physical and digital worlds^5,8^. This technology merges the immersive, computer-generated environments of VR with the integration and interaction of the real world found in AR. However, the terms MR and AR are often considered synonymous^9–11^. MR has the potential to revolutionize surgical interventions for diagnosis, planning, and surgical training^12^. By offering a dynamic and immersive environment, MR opens up new horizons for enhanced visualization, analysis, and strategic decision-making. However, the potential role of MR in preoperative planning for craniomaxillofacial trauma is yet to be thoroughly explored. Patient-Specific Implants (PSI) are currently being utilized to enhance understanding of a patient’s anatomy, facilitate surgical planning, and assist in the placement of orbital implants^10,13^.

This study aimed to evaluate the overall clinical applicability and performance of MR technology for preoperative planning, specifically focusing on patient-specific reconstruction of the orbit following orbital fractures. In addition to assessing the potential value for residents’ training, MR techniques were compared to conventional methods for diagnostic accuracy, planning workflow efficacy, and preoperative patient-specific reconstruction planning for orbital fractures. By investigating the user-friendliness of MR with an interface, this study sought to enhance the accuracy of PSI planning and optimize the entire preoperative process by proposing a new, more efficient workflow for orbital reconstruction.

## Materials and methods

### Study setting

This crossover study compared the MR approach to conventional 3D printing in the evaluation of maxillofacial traumatology diagnosis and treatment planning. A power calculation was performed using G*Power Analysis for the Wilcoxon signed-rank test (matched pairs), which revealed a sample size requirement of 16 participants for an effect size of 1 (mean of difference = 1 and standard deviation of difference = 1), power of 95%, and α value of 0.05.

The primary outcomes of this study are diagnostic accuracy, therapeutic planning advantages, efficacy of the evaluation method, and user-friendliness of MR in maxillofacial traumatology management. These outcomes were compared to conventional two-dimensional (2D) screen technology, with data collected and evaluated through survey questionnaires (refer to the questions presented in Table 1).

**Table 1:** Final results questionnaire.

Personal rating	MR (%)	3D (%)	Both (%)
Which method did you prefer?	56.25	37.5	6.25
Which method would you use again for an orbital fracture?	37.5	31.25	31.25
Which method was the easiest for diagnosis?	25	75	0
Which method was the easiest for trauma planning and review?	75	18.75	6.25
Which method was easiest to adjust or adapt to your planning?	62.5	31.25	6.25
In which method did you experience simulator sickness?	12.5	0	0
*In which method was/is this good/better?*			
3-dimensional perception	75	18.75	6.25
Visualization and depth	75	18.75	6.25
Intuitive control and examination of images	56.25	31.25	12.5
Overall user friendliness	56.25	18.75	25
Providing a 3-dimensional idea of the surgical/anatomic site	56.25	18.75	25
Display (resolution)of the structures and anatomical sites	81.25	18.75	0
Improvement of preoperative workflow	68.75	18.75	12.5
Review and adjustment of treatment plan	75	12,5	12.5
Evaluation of correct position of designed plate	50	37.5	12.5
Potential of (daily) clinical implementation	62.5	25	12.5
Interaction with other surgeons (hypothetically)	81.25	6.25	12.5
Learning curve	50	18.75	31.25
Mean global satisfaction score	80.6	72.5	/

Secondary outcomes encompassed analyzing time differences between approaches and assessing the potential effects on the learning curve, particularly for residents’ learning progression. This study was adhered to the principles of the Declaration of Helsinki, received approval from the Personal Data Protection Committee, and is registered under identification number S65739.

### Data collection and processing

The clinical records and radiographs of 10 patients who presented with a unilateral bony deficit of the orbital floor, roof, rim, and/or zygomatic complex between January 2014 and January 2020 were included in the study. The patient diagnoses and fracture specifications are presented in Table 2. All data was anonymized prior to inclusion for every case/patient in the study. Each patient had undergone a preoperative Computed Tomography (CT) scan and fulfilled the main criteria for surgery, which included eye motility problems, enophthalmos, and/or hypoglobus (vertical orbital dystopia). Additionally, the patients were at least 18 years old at the time of presentation. For each patient, a pre-and postoperative radiologic scan with 1 to 2 mm slice thickness was available. Patients with pre-existing or concurrent traumatic ophthalmic diseases or congenital craniofacial syndromes were excluded.

**Table 2:** Patient diagnoses and fracture specifications.

	AO Classification^21^ and side	Posterior ledge	Inferomedial angle	Fat prolapse/muscle entrapment	Other fractures
Case 1	Orbital floor (L)	Intact	Non-intact	Fat prolapse	Le Fort I + Zingg type C
Case 2	Orbital floor (L)	Intact	Non-intact	Fat prolapse	Zingg type B
Case 3	Orbital floor (R)	Intact	Non-intact	Fat prolapse	Le Fort I + NOE + Zingg type B
Case 4	Orbital floor (L)	Intact	Non-intact	Fat prolapse	
Case 5	Orbital floor + orbital medial wall (R)	Intact	Non-intact	Fat prolapse	
Case 6	Orbital floor (R)	Intact	Non-intact	/	
Case 7	Orbital floor + orbital medial wall (R)	Intact	Non-intact	Fat prolapse	Le Fort I
Case 8	Orbital floor + orbital medial wall (L)	Intact	Non-intact	/	
Case 9	Orbital floor + orbital medial wall (R)	Intact	Non-intact	Fat prolapse and muscle entrapment	
Case 10	Orbital floor + orbital medial wall (R)	Intact	Non-intact	Muscle entrapment	Zingg type B

R = right; L = left; Zingg type B fractures are tetrapod fractures; Zingg type C fractures are comminuted fractures^3^; NOE = naso-orbito-ethmoid fracture.

The study included 16 participants (maxillofacial residents with basic surgical experience). Informed consent was obtained from all participants prior to participation. From the 10 patient cases, the 5 cases for the MR method and the 5 cases for the 3D method were presented in a randomized order. Each case was evaluated in three phases: first diagnosis, then segmentation and planning, and lastly revision.

Segmentation was performed based on the standardized head orientation (Frankfurt horizontal plane). The orbit and zygomatic bone were automatically segmented bilaterally using Brainlab Elements (Brainlab AG, Munich, Germany). On each side, the orbit and zygomatic bone were merged, and then the unfractured side was mirrored, followed by a volumetric match on the bony healthy part of the fractured side.

**Fig. 1:**
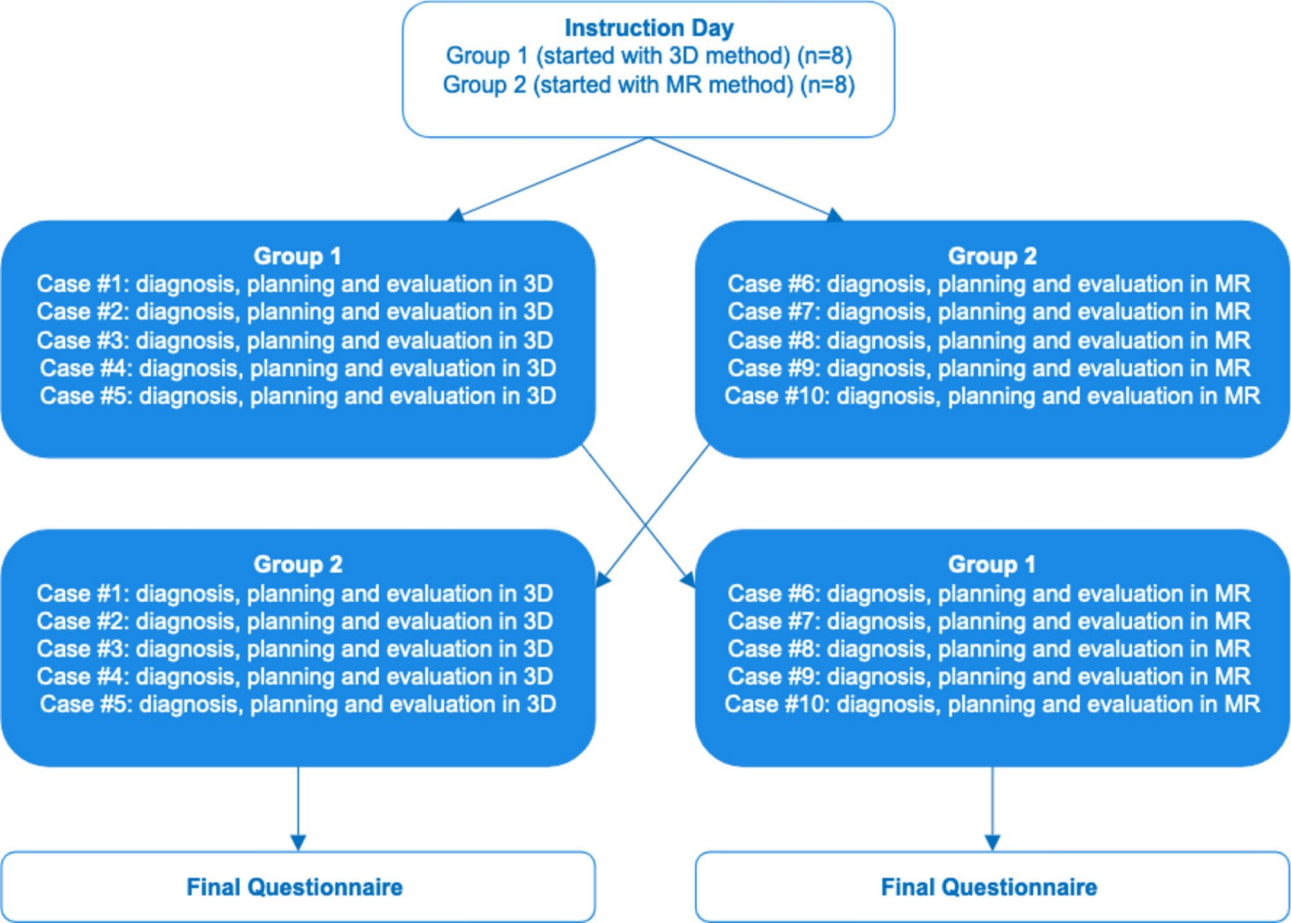
Crossover study design. (3D = 3D or conventional method; MR = mixed reality). The 5 cases for the MR method and the 5 cases for the 3D method were presented in a randomized order. (n = number of participants).

**Fig. 2:**
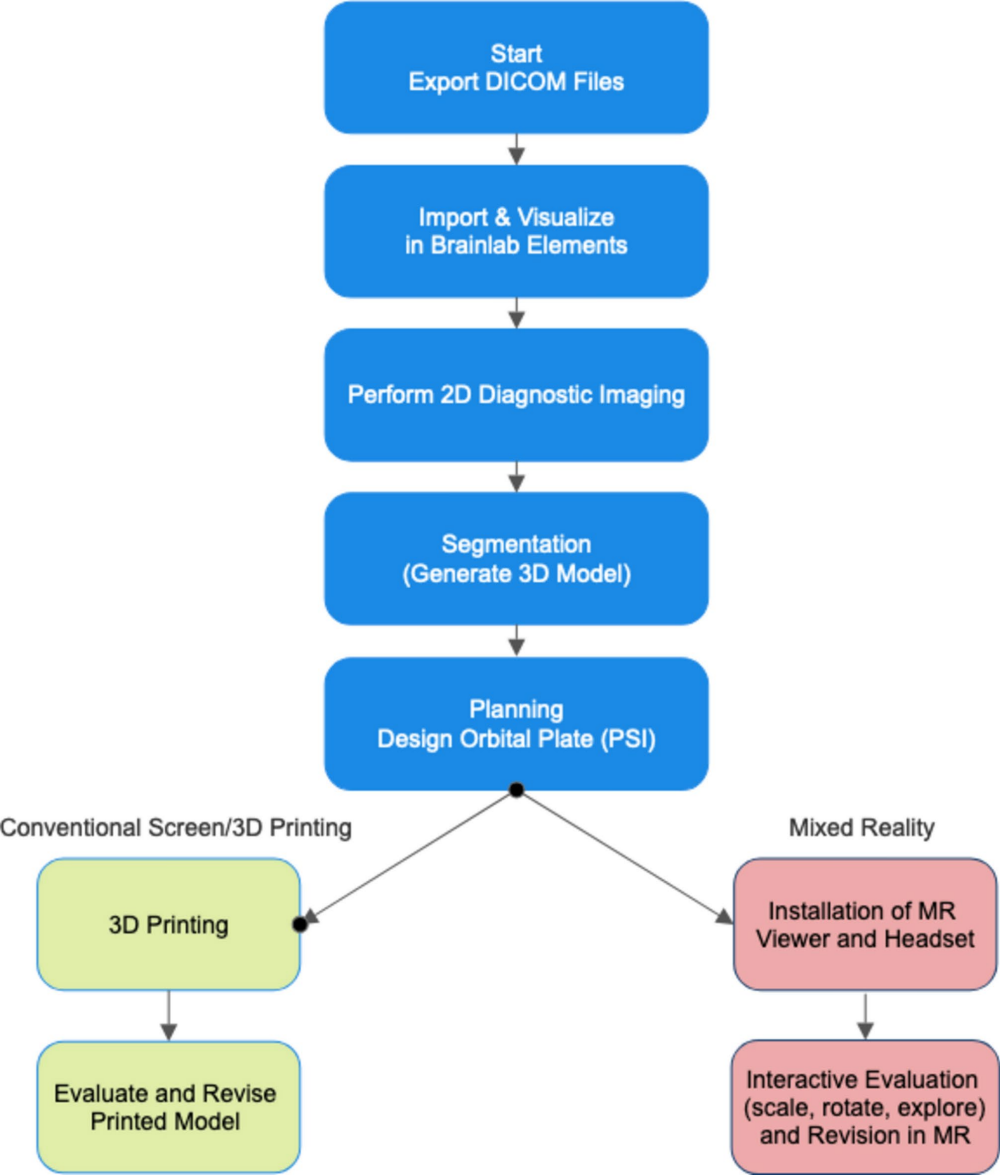
Flowchart illustration the key steps in the software’s planning procedure for both the conventional screen/3D printed method (left side) and the Mixed Reality Method (right side). Each step is color-coded to differentiate between shared, conventional, and mixed reality specific processes. (DICOM = Digital Imaging and Communications in Medicine; 2D = two-dimensional; PSI = Patient-Specific Implant; 3D = three-dimensional; MR = mixed reality).

Based on the crossover design (Fig. 1), Group 1 initiated the study with five cases utilizing the conventional screen method/3D printing approach. This process consisted of a Digital Imaging and Communications in Medicine (DICOM) export, import and visualization/diagnostic imaging in 2D using Brainlab Elements software (Brainlab AG, Munich, Germany), segmentation, design of the orbital implant, and evaluation of the 3D-printed model (printed by Objet Connex 350 [Stratasys, Eden Prairie, MN USA] with a layer thickness of 30 µm). On the other hand, Group 2 began with five cases in the MR approach. The MR procedure was identical, but instead of 3D printing, the bone model and orbital implant were visualized in Brainlab’s Mixed Reality Viewer (Brainlab AG, Munich, Germany). The MR headset (Magic Leap, Inc. [Plantation, FL, USA]) was connected via WiFi to the Brainlab Elements computer. In MR, the user saw the bone surface and the planned orbital implant as an interactive stereoscopic 3D model. With a handheld controller the user could move, scale and rotate the model. It was also possible to crop or walk inside the bone model to evaluate the fit of the orbital implant. The complete flowcharts for both methods are presented in Fig. 2.

Following both evaluations in MR and 3D printing, all participants completed a final questionnaire assessing user-friendliness and overall performance in visualization, interaction, segmentation, treatment planning, and evaluation of both methods (questionnaire presented in Table 1).

Furthermore, the time taken for the three phases (diagnosis, planning, and treatment evaluation) was recorded, and the accuracy of planning and user experience was further scrutinized. An expert evaluated the diagnostic accuracy and correctness of the planning. The bullet points used to calculate the total score for both the participant (self-evaluation) and the expert included posterior ledge support, medial and lateral bone support, (free) clearance of the inferior orbital fissure, reconstruction of the medial wall if indicated, correct fixation on the anterior rim, free tear duct, possibility of screw fixation, absence of optic nerve compression, absence of infraorbital nerve compression, and coverage of all fractures. Accordingly, a self-overestimation analysis was conducted using self-evaluation minus expert evaluation scores. Objective endpoints were evaluated blindly, while subjective endpoints underwent a double-blind evaluation process.

### Statistical analysis

A mixed model was used for data analysis, using a percentage as the response variable, and considering the method, group, and their interaction. This interaction captures the crossover effect, determining if the MR-3D difference is impacted by the method order. Participants and cases were treated as random effects in the analysis. The results are presented as means, mean differences with 95% confidence intervals, and an overall method effect corrected for the group.

Additionally, a linear mixed model was used for data analysis with diagnosis score (as a percentage) as the response variable, and method, group, and their interaction included in the explanatory model. Separate analyses were performed to analyze the learning curve in 3D and MR. Linear mixed models were also used for data analysis with planning time as the response variable, and case order, group, and their interaction as explanatory variables. Participants and cases were modeled as random effects. The results are presented as slopes with 95% confidence intervals, where the slope indicates the change in planning time with each additional case (learning curve).

Furthermore, differences in satisfaction scores between MR and 3D were calculated by comparing scores between groups. The crossover effect was tested by comparing the difference scores between both groups using the Mann–Whitney U test (Table 3). The overall effect of the method was tested using the Wilcoxon signed-rank test.

**Table 3:** Mean overestimation and difference between methods.

3D		MR			
Mean (95% CI)	*p*-value	Mean (95% CI)	*p*-value	Mean difference (95% CI)	*p*-value
1.163 (0.619;1.706)	< .0001	0.500 (−0.044;1.044)	0.0712	−0.662 (−1.252;−0.073)	0.0280

CI: confidence interval.

Mean > ( <)0: higher (lower) score for self-evaluation compared to expert evaluation.

Mean difference > ( <)0: higher (lower) over-estimation for MR compared to 3D.

A self-overestimation analysis was conducted by comparing self-evaluation with expert evaluation scores. The data analysis was performed using linear mixed models with self-overestimation as the response variable, and case order, group, and their interaction as explanatory variables. The interaction between case order and group captures the crossover effect, i.e., whether the learning curve depends on the order of the methods. Participants and cases were modeled as random effects. The results are presented as slopes with 95% confidence intervals, where the slope indicates the change in self-overestimation with each additional case (learning curve).

Linear mixed models were used to explore the effects of the year of training and case on various outcomes, providing slopes and 95% confidence intervals. Case difficulty was determined through z-scores for different scores and times. Linear mixed models were used for data analysis with case as a categorical explanatory variable, and a two-sample t-test was used to compare the difficulty of 3D and MR cases.

## Results

A total of 10 orbital trauma cases were included in the study, with 30% identifying as female and 70% as male. The average (± SD) age was 49.4 years (± 11.3). Sixteen residents participated in the workflow across the 10 orbital trauma cases, which included diagnosis, planning, and revision/evaluation. Consequently, a total of 160 planning sessions (80 in MR and 80 in 3D) were analyzed and compared.

### Diagnostic accuracy and time

The *p*-value for the crossover effect for diagnostic accuracy was *p* = 0.46, indicating no evidence of a crossover effect. Additionally, diagnosis scores did not differ between MR and 3D (*p* = 0.93).

The mean time (SD) for diagnosis using MR and the conventional screen method was 6′ 38″ (± 2′ 29″) and 5′ 58″ (± 2′ 26″), respectively. The *p-*value for this crossover effect was significant at *p* < 0.0001. Diagnosis time was shorter with MR in Group 1; however no significant difference was found between the two groups (*p* = 0.20). In Group 2, diagnosis time was significantly longer with MR (*p* = *0.003)*. Overall, MR and 3D did not differ (*p* = 0.37). The complete workflow times are presented in Table 4.

**Table 4:** Step-by-Step workflow timing of both methods.

Workflow	MR workflow time (mean)	Conventional (3D) workflow time (mean)	*p*-value	Crossover effect
1. Diagnosis	6′ 38″ (SD ± 2′ 29″)	5′ 58″ (SD ± 2′ 26″)	0.37	Yes (*p* < .0001)
2. Planning	15′ (SD ± 3′ 02″)	14′ 25″ (SD ± 4′ 10″)	0.25	Yes (*p* < .001)
*Learning curve MR*				Yes (*p* = .007)
*Learning curve 3D*				No (*p* = .054)
3. Printing	/	59′ 48″ (SD ± 11′ 9″)		
4. Revision/evaluation	2′ 10″ (SD ± 40″)	1′ 49″ (SD ± 37″)	0.16	Yes (*p* = .0002)
*Learning curve MR*				Yes (*p* = .019)
*Learning curve 3D*				No (*p* = .29)
Total time (mean)	23′48”	1h22′00”		

MR = mixed reality; 3D = three-dimensional or conventional method.

### Planning time and learning curve

The mean time (SD) for planning in MR and the conventional screen method was 15′ (± 3′ 02″) and 14′ 25″ (± 4′ 10″), respectively. A significant crossover effect was observed for planning time (*p* < 0.001), with planning time being significantly shorter with MR in Group 1 (*p* = 0.0003*)*. In Group 2, planning time was significantly longer with MR (*p* < 0.0001). Overall, MR and 3D did not differ *p* = 0.25).

The mean printing time (only for the conventional 3D workflow) for the orbit was 59′ 48″ (± 11′ 9″) minutes. The complete workflow times are presented in Table 4.

**Fig. 3:**
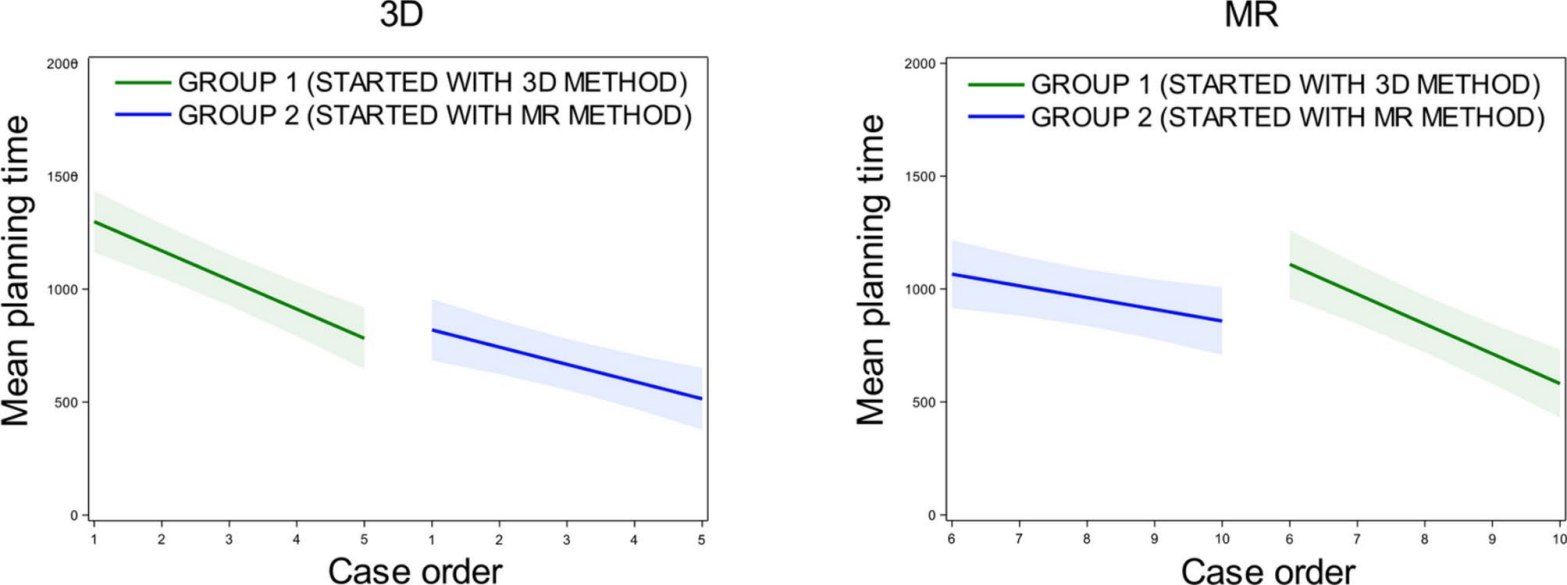
Learning curves for planning time in 3D (left) and MR (right). Negative slopes indicated a decrease in planning time (higher learning curve) over case order. Slope (95% CI) for group 1 in 3D and MR is −129.2 (−167.3;−91.2) and −132.0 (−173.3;−90.8) respectively. Slope (95% CI) for group 2 in 3D and MR is −76.3 (−114.4;−38.2) and −51.9 (−93.4;−10.4) respectively. (CI = confidence interval).

In the context of the learning curve for planning (Fig. 3), a crossover effect was observed for MR (*p* = 0.007*)*, although the effect was borderline insignificant for 3D (*p* = 0.054). Negative slopes indicated a decreased planning time (higher learning curve) over case order.

### Revision/evaluation time and learning curve

The mean time (SD) for revision or evaluation in MR and 3D printing was 2′ 10″ (± 40″) and 1′ 49″ (±37″), respectively. A crossover effect was found for revision time (*p* = 0.0002). No difference in revision time was noted with MR in Group 1 (*p* = 0.56). However, a significantly longer revision time with MR was found in Group 2 (*p* < 0.0001*)*. Averaged over both groups, the revision time was significantly longer with MR (*p* = 0.016). The complete workflow times are presented in Table 4.

**Fig. 4:**
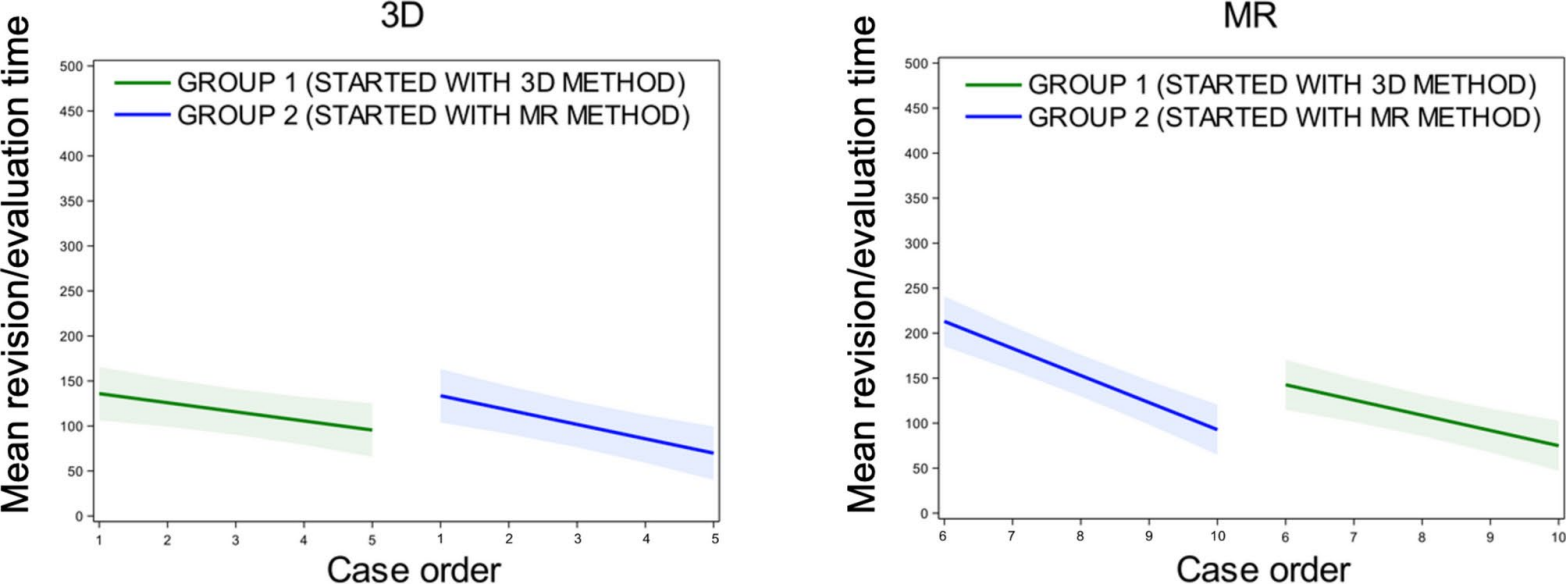
Learning curves for revision/evaluation time in 3D (left) and MR (right). Negative slopes indicated a decrease in revision time (higher learning curve) over case order. Slope (95% CI) for group 1 in 3D and MR is −10.1 (−17.8; −2.4) and −16.9 (−24.7; −9.2) respectively. Slope (95% CI) for group 2 in 3D and MR is −15.9 (−23.7; −8.2) and −30.1 (−37.8;−22.3) respectively. (CI = confidence interval).

In the context of the learning curve for the revision or evaluation (Fig. 4), a crossover effect was found for MR (*p* = 0.019) but none for 3D (*p* = 0.29). Negative slopes indicated a decreased revision time (higher learning curve) over case order.

### Satisfaction score

The mean global satisfaction scores for MR and 3D were 80.6% and 72.5%, respectively. A crossover effect was not evident (*p* = 0.23*)*. A significant difference was observed, indicating higher overall satisfaction with MR compared to 3D (*p* = 0.014). In 56.25% of the cases, the MR method was preferred. However, for diagnosis, most participants (75%) preferred 3D over MR. The results are depicted in Table 1.

### Correctness of the planning/self-overestimation

The results do not suggest crossover effects (MR *p* = 0.41; 3D *p* = 0.61) or learning curves (*p* > 0.05).

In the absence of learning curves, the average self-overestimation in 3D and MR was estimated, along with the difference between both methods. Table 3 reveals a significant self-overestimation among participants when using 3D models (*p* < 0.001). Specifically, the self-evaluation scores were significantly higher than the scores assigned by expert reviewers, suggesting that participants perceived their planning accuracy with the 3D-printed models to be better than it actually was. Furthermore, an insignificant overestimation was found in MR (*p* = 0.0712). Additionally, the overestimation was significantly higher in 3D compared to MR (*p* = 0.0280).

### Effect of year of training/effect of case difficulty

The study results did not suggest an effect of the year of training on the different scores (*p* > 0.05). Similarly, the difference between the two methods regarding case difficulty was not discernible (*p* > 0.05).

### Simulator sickness

During the study, two participants experienced simulator sickness while using MR technology.

## Discussion

The purpose of this clinical crossover study was to enhance the preoperative planning of orbital reconstruction by addressing the complexities and limited intraoperative visibility encountered during surgical procedures. Traditional approaches may fall short in overcoming these challenges^10^. This study explored the innovative application of MR in preoperative planning to streamline preoperative workflow and improve surgical outcomes.

The research findings demonstrated a reduced workflow for planning a PSI for orbital fractures using Brainlab Elements with the integration of MR. Although the revision time in 3D is significantly shorter due to the headset installation time, the complete workflow includes approximately one additional hour of printing time, making MR planning substantially quicker than the conventional 3D method. Furthermore, the study assessed the accuracy of MR planning, revealing a significant overestimation in 3D compared to MR. This indicates that maxillofacial surgeons perceived their planning to be more effective than it actually was when using the 3D-printed models. In contrast, with MR, fewer mistakes were made, resulting in higher accuracy in planning validation. Similar advantages of MR were noted in orthognathic surgery planning, where MR improved the accuracy of virtual occlusion settings while reducing the time and errors associated with traditional dental models and 3D-printed casts^14^. Another study similarly demonstrated that VR technologies streamline planning and enable surgeons to perform tasks more efficiently, mirroring the findings of this study, which show that MR enhances workflow efficiency and accuracy in surgical planning^15^.

Moreover, both planning and revision exhibit a significant learning curve. A crossover effect was observed only for planning, indicating that the group that started with 3D, experienced a more pronounced learning curve in both 3D and MR technologies. This learning process aligns with findings from other studies that also highlight the steep learning curves associated with VR and MR modalities^5,6,14,15^. Nevertheless, effective training modules are crucial for assisting users to acclimatize to these technologies^9,15^.

Given these advantages and the potential for advancements in surgical planning, the overall satisfaction score for MR was higher than that for 3D. This preference was primarily attributed to improved 3D interpretation, enhanced visualization and depth perception, superior resolution of structures and anatomical sites, higher quality of interaction with other surgeons, improved workflow and better review and adjustment of treatment plans. Existing literature confirms that these improvements in 3D interpretation and visualization significantly contribute to the effectiveness of MR in surgical contexts while also meeting clinical precision requirements^10,16^. Nonetheless, most participants still preferred the 3D or conventional methods for diagnosis over MR.

**Fig. 5:**
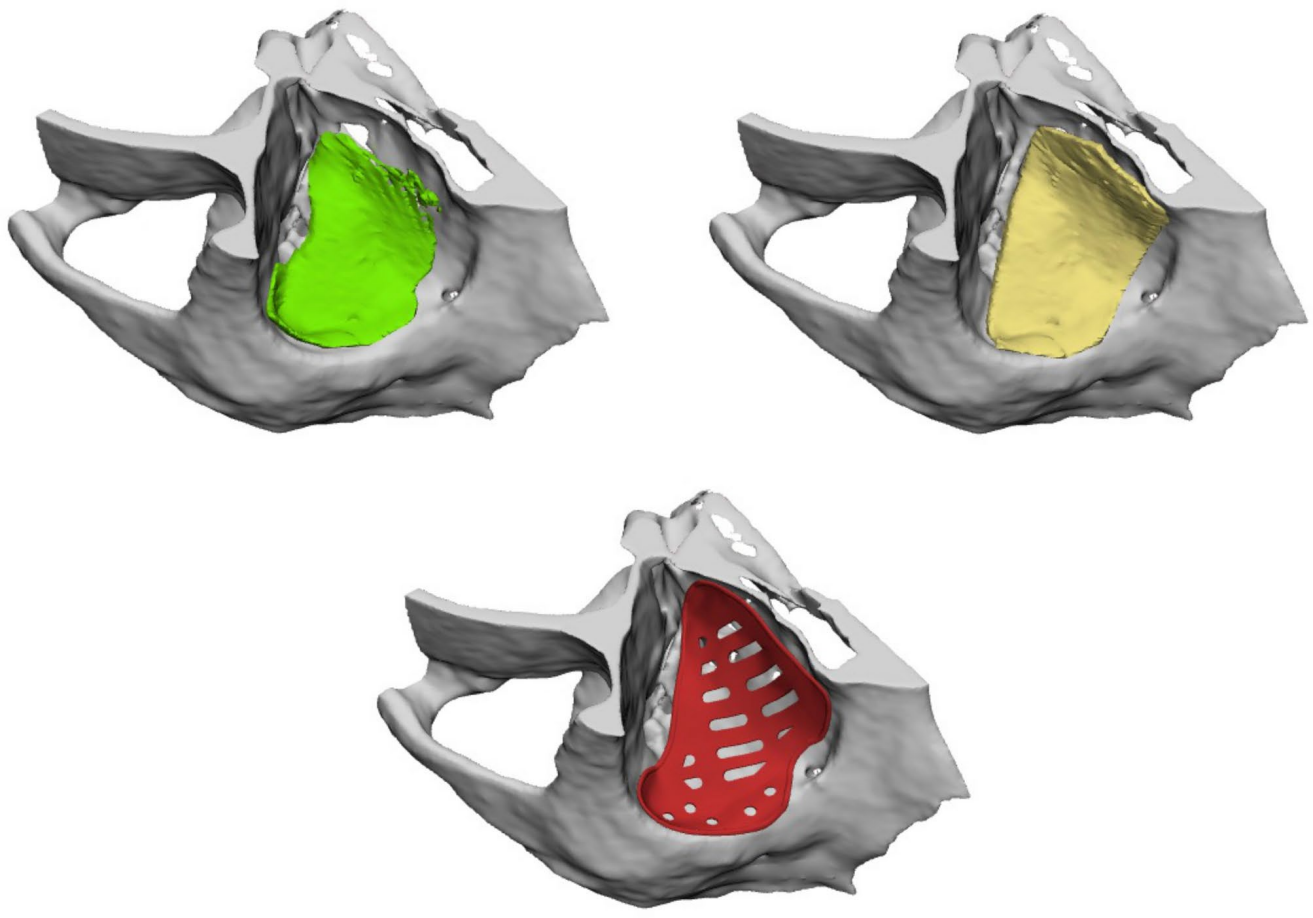
Example of PSI for orbital fractures designed by two different participants in Brainlab Elements (green and yellow) versus the finished fine-tuned implant designed by an engineer (red). Expert-score green design = 10/10, expert-score yellow design = 9/10. (PSI = Patient-Specific Implant).

The outcomes of this study suggest that MR has the potential to revolutionize orbital reconstruction (Fig. 5), presenting an opportunity to enhance surgical visualization and planning and ultimately improving patient outcomes. Its ability to transform complex medical images into interactive and realistic 3D models has far-reaching implications for both healthcare professionals and patients. Moreover, MR facilitates a detailed examination of fractures by magnifying and manipulating intricate details while eliminating obstructive elements. Unlike conventional 3D-printed models that are restricted by anatomical constraints, MR offers unparalleled flexibility, enabling the rotation of structures, manipulation of individual segments and comprehensive disassembly of images^14^. This immersive experience within the MR headset surpasses traditional models, thereby enriching reconstructive precision.

However, one limitation of MR is its reduced field of view and display resolution. The computing power can affect the quality and immersion during critical moments. Simulator sickness is another recognised limitation of VR/AR. However, one study noted that simulator sickness may occur less frequently in MR compared to VR/AR^17^.

Based on the existing literature, MR technology has been successfully implemented for preoperative planning in orthognathic surgery, maxillofacial reconstruction, and the tracking of maxillofacial nerves and vessels^12,18^. Anticipating future developments, the incorporation of MR for intraoperative navigation could represent a crucial advancement. Moreover, a key difference from existing intraoperative guidance systems is that, with traditional navigation, surgeons can only confirm their positioning. Conversely, by utilising MR technology, they can view a transparent representation of the patient, allowing for precise guidance to the exact area of interest^10,19^. Additionally, MR can help alleviate the challenge of mentally superimposing the planning information onto the patient during surgery^19,20^. Lastly, current navigation systems do not adequately address soft tissue management^9^. However, emerging techniques such as MR show great promise in globe positioning, orbit volume restoration and soft tissue management^9,18^.

## Conclusion

MR and 3D techniques require an equal amount of time for diagnosis, planning, and treatment evaluation, but 3D techniques require a significant additional time investment for model printing. Consequently, when designing a PSI, MR facilitates a faster workflow while also eliminating the additional costs associated with 3D printing. This combination suggests potential cost savings in the long run, although further research is required to directly evaluate cost-effectiveness. Moreover, according to the participants, MR allows for better visualization and awareness of the injury, which may lead to more successful outcomes in restoring the integrity and functionality of the bony orbit.

## Data Availability

The datasets used and/or analyzed during the current study available from the corresponding author on reasonable request.
